# A Few Flaws in Dentistry

**Published:** 1906-10

**Authors:** J. R. Osborne

**Affiliations:** Shelby, N. C.


### A FEW FLAWS IN DENTISTRY.

Dr. J. R. Osborne, Shelby, N. C.

Flaw-picking is a modern word in dentistry, but come to think about it, it is something we all do a great deal in our work. As a general thing it is no trouble to detect flaws—plenty of them—in natural teeth, but sometimes it requires close attention and tact to pick them out in time.

I have been thinking for some time that there are some little rough places in the profession—a screw or two that might be tightened a little—a few flaws that should be picked, and by your permission and the rope extended by the committee, I proceed to attend to this this morning in this way as best I can—as I see it.

It seems to me, and has for some time, that the dentist does not receive the recognition he deserves. We are right important folks, and are generally looked upon with about the same welcome as the tax-collector. I would suppose the average patient thinks as reverently of the dentist as he does of some one to whom he owes forty dollars and don't ever intend to pay. Dentists, as a rule, when it comes to appreciation of services, have about the same number of friends as an old blind fox hound. I hope I have not over picked this flaw. If the young dentist who has spent his money, pored over his books as he should, stood the "skeery" test before the board, borrowed money to pay for his instruments, and is trying in every honorable way to build up a legitimate practice, can think he is properly recognized by the laity, when most of his patients—the ones he thought would never forsake him—will step down to some jackleg who has about the qualifications for practicing denistry that my voice has for opera singing; if that young fellow can think that folks look upon him as they should, he is optimistic a plenty.

Of course, we have no way of making folks do our way—there's no rope around them and they will continue to go where they please—and any paper read or discussion carried on here will not change the trend of the human mind on this line. I am not offering a panacea—just flaw-picking a little this morning. A little later on if I don't forget it, I mean to try a poultice.

I want to touch next upon the dentist who is in the work for the dollar only. Don't think what I say will change or make him useful. It can hardly be done. Just flaw-picking—that's all. This fellow may have the mechanical tact and be able to answer the questions required of him by faculty and board, and of course has all the rights, legal and otherwise, but what I wish to emphasize is this: The fellow who is in it for the dollar only, and makes no move but the one that points to the greasing of his weasel skin, never does the profession any good except it be in an incidental way. He will never join a dental society or attend one unless it comes close and then to criticise. He never writes for the journals—takes up too much of his valuable time. As for testing materials and tinkering with tools and appliances, he will be sure to wait until some other fellow makes the sacrifice of time and money and establishes something standard or definite. The dentist who is willing to abstract so much from his profession and give nothing in return is a rascal and a helper in fostering the idea that is too universally prevalent—that the dentist is a tradesman.

What about the young man who is thinking of taking up dentistry who has no mechanical tact? That is a bad old flaw. It seems to me a miscarriage of common sense for a young man to fail to see this early in the work. There is no calling that demands a more decided turn to mechanics than ours. When a young man calls on me and wants my advice about his taking up dentistry, I try to impress him very forcibly on this point. I tell him if I could see a chicken coop of his manufacture, I could pass on that point more definitely. I want the coop to look well and to be handy and convenient for the busy, over-worked hen. This is a flaw easily found and hard to remedy. Hard to make a race horse out of a mule, and what I am saying about him is not to tear him down or discourage him. Just flaw-picking a little this morning.

This brings me to the fellow who joins the Society to be unanimously elected president in not more than five years after joining. Of course the thing could not run longer than that without him at the head. It might collapse or coalesce with something. His air, dress, pomposity, knowledge of parliamentary law, hair greese and dignity are things the Society can't get along without. Neglecting to choose him for chief, the Society loses him forever and as soon as he "unjines" he commences to cuss. The fusing point of his dignity and self-importance has been reached and he moulds his alloy into a pruning-hook and proceeds to go around "gougin" a gash in everything that pops its head up that looks the least bit professional. Nature and the Dental Society here join hands in bringing out a full fledged fool. Prod a fool and you have a bigger fool than ever. I hope you catch the gist of this flaw and the only reason I bring it out is that it may stimulate us all to be better Society workers and be better contented.

I want to flaw-pick on Ethics. It is about as harmless a concern as I know of. If it learns many of us the science of human duty to any extent it is worth the money. The way I act towards other folks—dentists and all—was pretty well settled before I knew there's a difference in dentistry and sheep-shearing. The good Lord has a good deal more to do with that than anybody, and what few touches are put upon it by the influence of the mother, home and fireside, and the environments of early life, are about all that amount to anything. Making a gentleman out of a jackass after he has passed the examining board is an up-hill business. As a success, making whistles of pig's tails beats it all hollow. Every dentist is the architect of his own ethics, and you can always get at it exactly if you can find out the estimate he places upon the Golden Rule. "Fun is for the millions—ethics for the few," said old Billings, and I suppose he meant there are more folks in the world who enjoy the ludicrous than there are who are willing to act right toward other people.

But lets flaw-pick again. We need some things to housekeep with that we have not got. For instance, we go wild over our praise and prescription of hot air, but haven't we a way of getting it so it would be hotter and more continuous? A little pump-gun with a knot on the end to be stuck in a flame till it heats and then stand there and pump a little every once in a while. That's pursuing a scientific idea with a whizz, ain't it? Why don't some of us who are able to get up an apparatus that will do this effectively? We need hot air, uncontaminated air, and distilled water, and we don't want it in squirts—we want it to come continuous and with force. And if there is so much in aseptic dentistry, and I believe there is, we would be getting the benefit of that kind of practice. We talk of germs of disease and how easy it is for them to propagate and to disseminate their heltry and how important it is for us to use every precaution to destroy the little rascals and their foothold for colonization—and not a single method of this kind to make them even catch a short breath.

Let me flaw-pick a little more and I am done. "Ain't we a mess" when it comes to filling a root canal? Don't you think we ought to standardize a little on this line? Suppose an intelligent patient of ours should pick up a dental journal and open at "root canal filling." After reading the article he asks for more literature on the subject and you give him access to your library and he reads on until he has exhausted the theme. What do you suppose his idea of dentistry is to the members of it being a unit in their work? One member of the profession swearing that chlora-percha is the only thing that will do for his work, and another says put me down for gutta-percha points, another uses a creosoted stick, another charcoal points, another gold, another even alloy, another iodoform, another beeswax, another carbolic acid and cotton, and so on without end; everything in the vegetable and mineral kingdoms. I declare, I think we ought to get down on this in not quite so scattered a position. But I am not here to tell you how. Just flaw-picking a little this morning.

All I mean in what I have said is that we all do the best we can for ourselves and the profession. We all have faults as men and as dentists, flaw-picking or no flaw-picking. You might as well look for a perfect horse as perfection in humanity. My highest desires are that we as members of a dental society and brethren of a noble calling go forward in our work and in our acts toward each other, and in our treatment of the good people we serve. Make us more brotherly, useful and dignified. The hope of dentistry is in organization. I believe in local organization, and if each member would do all that he could to make each succeeding meeting better than the last one, there lies before us some great possibilities. I sometimes think that we lay members are not quite willing enough to help pull the old thing along as it should be. There is a lot of sacrifice and oceans of hard work to be done in keeping our

Society in line as it is and has been, and the more each one of us does the less it makes to be done by those who are generally looked upon to do it all. I flaw-pick no more.

